# Defining the correlates of lymphopenia and independent predictors of poor clinical outcome in adults hospitalized with COVID-19 in Australia

**DOI:** 10.1038/s41598-024-61729-5

**Published:** 2024-05-15

**Authors:** Priyanka Hastak, Deborah Cromer, James Malycha, Christopher R. Andersen, Eamon Raith, Miles P. Davenport, Mark Plummer, Sarah C. Sasson

**Affiliations:** 1 The Kirby Institute, University of New South Wales, Sydney, Wallace Wurth Building (C27), Cnr High St & Botany St, Kensington, NSW 2052 Australia; 2 Royal Adelaide Hospital, Adelaide, SA Australia; 3 University of Adelaide, Adelaide, SA Australia; 4 The George Institute for Global Health, Sydney, Australia; 5 Royal North Shore Hospital, Sydney, Australia

**Keywords:** Lymphopenia, COVID-19, Mortality, Hospital length of stay, Intensive care unit (ICU), Immunology, Biomarkers, Diseases, Health care, Medical research, Risk factors

## Abstract

Lymphopenia is a common feature of acute COVID-19 and is associated with increased disease severity and 30-day mortality. Here we aim to define the demographic and clinical characteristics that correlate with lymphopenia in COVID-19 and determine if lymphopenia is an independent predictor of poor clinical outcome. We analysed the ENTER-COVID (Epidemiology of hospitalized in-patient admissions following planned introduction of Epidemic SARS-CoV-2 to highly vaccinated COVID-19 naïve population) dataset of adults (N = 811) admitted for COVID-19 treatment in South Australia in a retrospective registry study, categorizing them as (a) lymphopenic (lymphocyte count < 1 × 10^9^/L) or (b) non-lymphopenic at hospital admission. Comorbidities and laboratory parameters were compared between groups. Multiple regression analysis was performed using a linear or logistic model. Intensive care unit (ICU) patients and non-survivors exhibited lower median lymphocyte counts than non-ICU patients and survivors respectively. Univariate analysis revealed that low lymphocyte counts associated with hypertension and correlated with haemoglobin, platelet count and negatively correlated with urea, creatinine, bilirubin, and aspartate aminotransferase (AST). Multivariate analysis identified age, male, haemoglobin, platelet count, diabetes, creatinine, bilirubin, alanine transaminase, c-reactive protein (CRP) and lactate dehydrogenase (LDH) as independent predictors of poor clinical outcome in COVID-19, while lymphopenia did not emerge as a significant predictor.

## Introduction

The COVID-19 pandemic caused by the SARS-CoV-2 virus has led to ~ 6 million deaths worldwide^1,2^. Early in the pandemic it was recognised that patients with severe disease often displayed low circulating lymphocyte counts. In data from Wuhan during 2020, it was reported that dividing patients infected with SARS-CoV-2 according to their clinical outcome revealed stratified lymphocyte counts, with those that died having profoundly low circulating lymphocyte counts (< 5% of leukocytes) and those who survived having lymphocytes in the normal range (> 20% of leukocytes)^3^. A meta-analysis of 3099 COVID-19 patients from 24 studies in 2020 confirmed that a lymphocyte count below the lower limit of normal was significantly associated with admission to ICU, ARDS (acute respiratory distress syndrome) and death^4^. Similarly, longitudinal studies in Korea and the USA demonstrated that lymphopenic patients with severe COVID-19 pneumonia had higher rates of ICU admission and death^5,6^.

COVID-19-associated lymphopenia has been associated with male, sex age > 55 years and the presence of medical co-morbidities including diabetes, hypertension, pulmonary disease, malignancy, cardiac, renal, pulmonary, and hepatic disease^7–9^. A study that examined lymphocyte counts and comorbidities found significantly lower lymphocyte counts in patients with at least one medical comorbidity compared to patients with no comorbidities^10^.

Lymphocytes are comprised of T cells, B cells, NK cells and their subtypes. T and B cells are key players in the adaptive immune response, facilitating cell-mediated and humoral immunity, respectively. The underlying physiological and molecular mechanisms driving the low lymphocyte counts in COVID-19, or indeed other forms of sepsis, are not well understood. COVID-19-associated lymphopenia may be driven by a reduction in bone marrow and/or thymic output, or an increase in trafficking and sequestration of lymphocytes to infected inflamed tissue, or lymphocytes may undergo high rates of activation-induced death and apoptosis^11^.

Given lymphopenia is a common laboratory feature of COVID-19 and is associated with development of ARDS, admission to ICU and death we aimed to define what demographic and clinical comorbidities were associated with lymphopenia by analysing data from consecutive adult patients hospitalised for the management of COVID-19. Additionally, we aimed to determine for the first time if lymphopenia was an independent predictor of length of stay, ICU admission or mortality.

## Methods

### Data collection and selection

The Epidemiology of hospital iNpatienT admissions following planned introduction of Epidemic SARS-CoV-2 to a highly-vaccinated COVID-19 naïve population in South Australia (ENTER COVID) registry was established to capture demographic, physiological, laboratory, treatment and outcome variables of all adult patients with real time polymerase chain reaction (RT-PCR) test-confirmed SARS-CoV-2 infection admitted to hospital for > 24 h [Central Adelaide Local Health Network Research Ethics Committee (project number 15809)]. This research was performed in accordance with the “Declaration of Helsinki”. We analysed the ENTER-COVID dataset for all admissions between November 2021 and February 2022 (N = 1547). For downstream analysis we only included patients admitted to hospital primarily for the management of COVID-19 and who swabbed positive for COVID-19 on admission (N = 1020). The platform for COVID-19 detection was the Bio-Rad CFX384 PCR Detection System (Bio-Rad Laboratories Pty Ltd, South Granville Australia). A positive sample was confirmed by either an E gene or N gene cycle threshold < 40. Viral loads were not calculated. We excluded patients admitted to hospital for an alternative diagnosis who incidentally swabbed positive for SARS-CoV-2. We also excluded patients who initially tested negative for SARS-CoV-2 on admission and then contracted a hospital-acquired COVID-19 infection. We set cut-offs for clinical laboratory parameters, where outlier values could not be reasonably attributable to COVID-19 (see Supplementary Table 1) and excluded patients above these cut offs. Following this exclusion, 811 patients were included for analysis.

The clinical outcomes of interest were (i) hospital length of stay (LOS), that was defined as number of days that a patient remained in hospital after a single admission event^12^. (ii) ICU admission, defined as a patient who spent a minimum of two hours in the ICU^13^. (iii) in-hospital mortality or death, defined as patients who died while admitted to the hospital^14^.

### Statistical analysis

We performed statistical analysis on clinical laboratory parameters categorized as continuous variables, while comorbidities (classified according to APACHE score^15^) and demographics were categorized as categorical variables. For categorical variables, differences between groups were determined by the Fisher’s exact test. For continuous variables, differences between groups were determined using two sample unpaired t-tests. Median values and interquartile ranges (IQR) are presented throughout. A p-value < 0.05 was considered statistically significant. Multiple comparisons were accounted for by using the Holm-Bonferroni method^16^. Relationships between lymphocyte counts and continuous variables were determined by Pearson’s correlation.

Multivariate regression analysis was used to determine the relationship between baseline demographics, biomarkers, and co-morbidities and lymphopenia as well as other outcomes of interest. Stepwise forwards regression was used to determine a multivariate model in which all predictor variables were significant, with the p-value for inclusion set at 0.05.

### Ethics declarations

The Human Research Ethics committee (HREC) and the National Health and Medical Research Council (NHMRC) has approved this study, data collection and analysis of the dataset at the Central Adelaide Local Health Network.

### Consent to participate and publish

Informed consent was obtained from all subjects in this study. The dataset was de-identified with consent to publish.

## Results

### Lymphopenia and demographics

**Figure 1:**
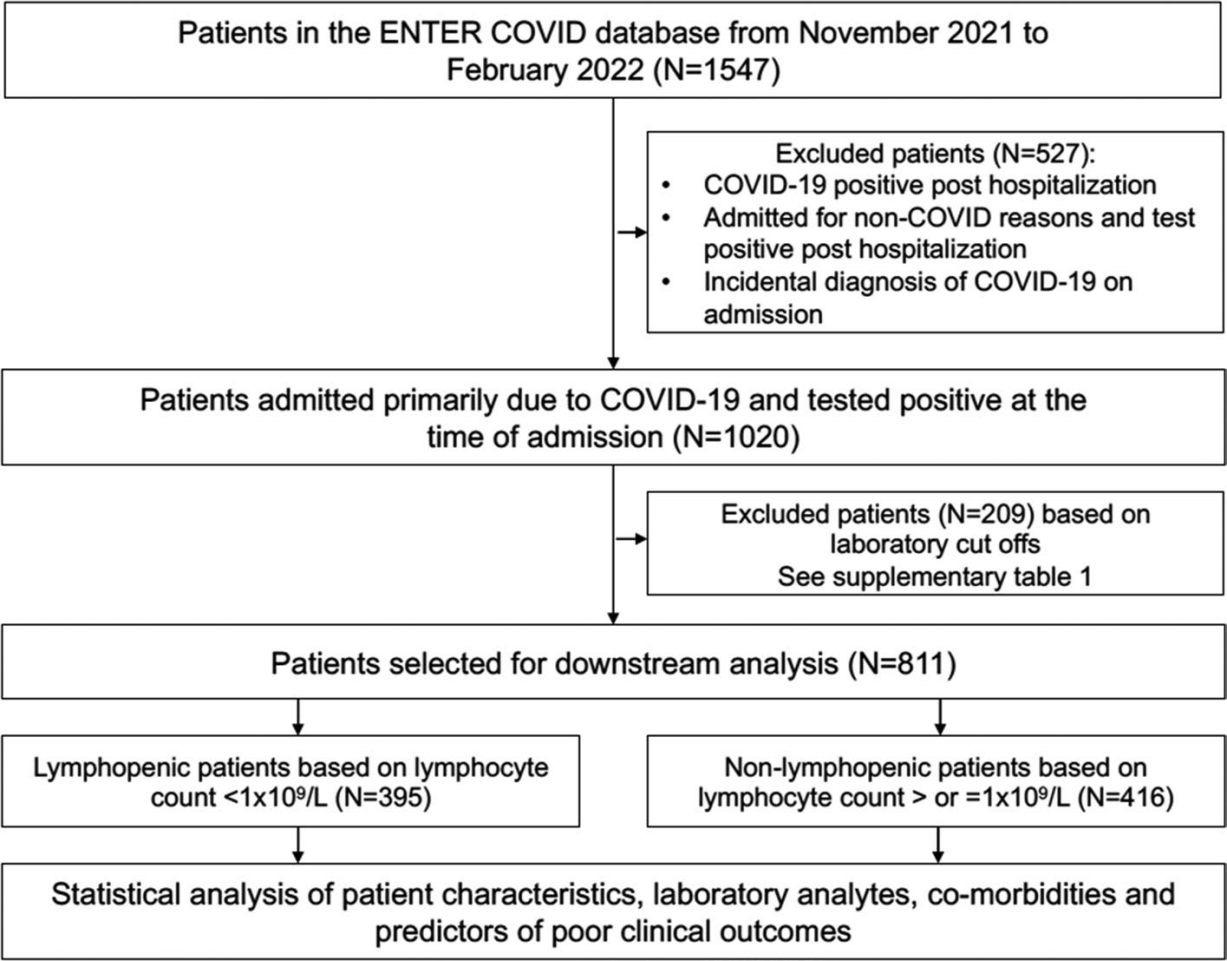
Flow diagram of selection criteria and cut offs for the ENTER-COVID dataset.

A total of 811 patients were analysed, of these, 395 were lymphopenic (absolute lymphocyte count < 1 × 10^9^/L) and 416 were non-lymphopenic on admission (Fig. 1).

Lymphopenia was over-represented in males (52%) and in people > 65 years of age (58%; median = 70 years). Patients with lymphopenia had a higher death rate (5.5% versus 2.2% p = 0.016) compared to non-lymphopenic hosts (Table 1). There was no significant difference in COVID-19 vaccination status (2nd dose or 3rd dose) between lymphopenic and non-lymphopenic patients (Table 1).

**Table 1:** Patient characteristics and associated co-morbidities.

	Lymphopenic (N = 395)	Non-lymphopenic (N = 416)	P-value (odds ratio)
Age (years), median (IQR)	70 (median); 25–95 (IQR)	65 (median); 23–78 (IQR)	
Patients > 65 years	229 (58%)	197 (47%)	**0.0025 (1.27)**
Men	207 (52%)	186(45%)	**0.0284 (1.36)**
Received 2nd COVID-19 vaccine	253 (64%)	257 (62%)	0.5136 (1.10)
Received 3rd COVID-19 vaccine	100 (25%)	110 (26%)	0.7486 (0.44)
ICU admission	40 (10%)	27 (6%)	0.0735 (1.62)
Death	22 (5.5%)	9 (2.1%)	**0.0160) (2.67)**
Smoking	19 (5%)	22 (5%)	0.873 (0.91)
Asthma	54 (14%)	84 (20%)	**0.015 (0.63)**
Diabetes	109 (27%)	116 (28%)	0.938 (0.98)
Hypertension	187 (47%)	154 (37%)	**0.004 (1.53)**
Chronic pulmonary disease	67 (17%)	67 (16%)	0.779 (1.64)
Chronic cardiac disease	104 (26%)	89 (21%)	0.117 (1.31)
Chronic liver disease	14 (3%)	12 (3%)	0.691 (1.24)
Chronic kidney disease	62 (15%)	51 (12%)	0.187 (1.33)
Chronic neurological disease	46 (12%)	47 (11%)	0.913 (1.04)
Hepatitis B	7 (1.7%)	6 (1.2%)	0.784 (1.23)
Hepatitis C	5 (1.2%)	8 (1.9%)	0.576 (0.65)
HIV	1	0	> 0.999 (0)
Tuberculosis	3 (0.7%)	3 (0.7%)	> 0.999 (1.05)
Malignant neoplasm	53 (13%)	45 (10%)	0.281 (1.28)

p values < 0.05 are shown in bold. IQR = interquartile range.

### Lymphopenia and co-morbidities

**Figure 2:**
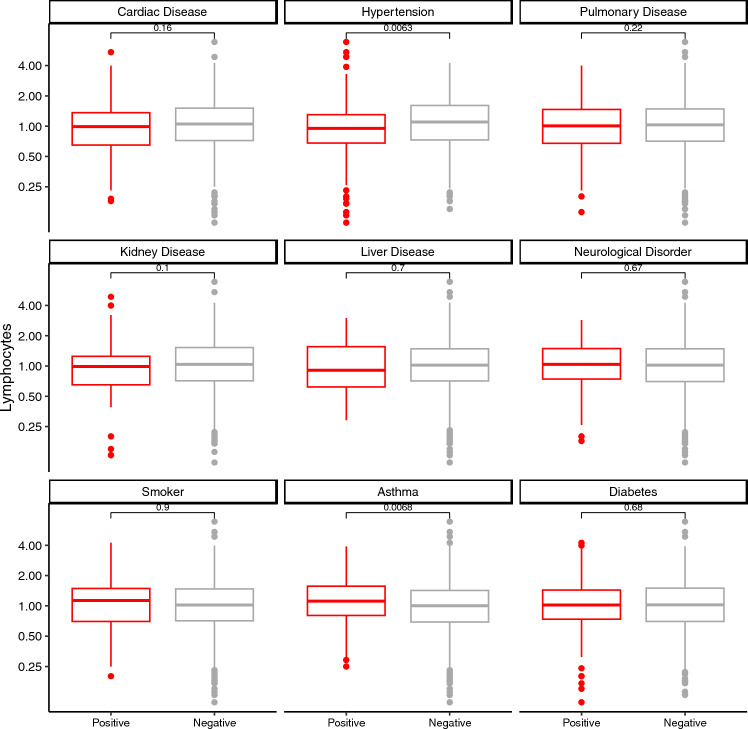
Lymphocyte counts and co-morbidities. Patients with hypertension had statistically lower median lymphocyte counts and those with asthma had statistically higher median lymphocyte counts. Grey = non-lymphopenic and red = lymphopenic patients.

Regarding medical co-morbidities, the rate of hypertension was higher in lymphopenic patients as compared to non-lymphopenic patients (47% versus 34% p = 0.004). There was no statistically significant difference in lymphocyte counts in patients with or without diabetes, active smoking, chronic pulmonary disease, chronic cardiac disease, or chronic liver disease, tuberculosis, malignant neoplasm (Fig. 2 and Supplementary Fig. 1). Of note, patients admitted to hospital for the treatment of COVID-19 with a past medical history of asthma were less likely to present with lymphopenia (odds ratio 0.63; p = 0.015; Table 1) although this difference did not retain statistical significance after accounting for multiple comparisons.

### Effect of lymphopenia on clinical outcomes

**Figure 3:**
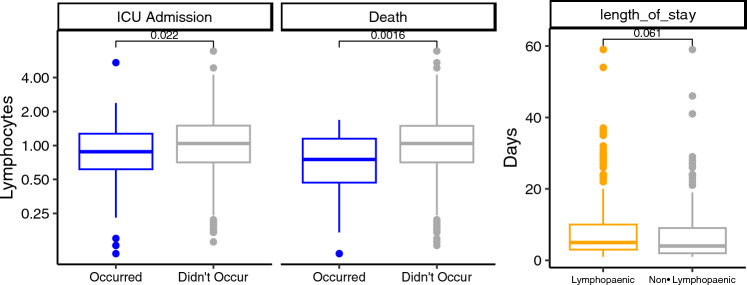
ICU admission and death were associated with lower median lymphocyte counts. Note that length of stay was only considered for stays of at least one day, and less than 100 days.

We assessed if lymphopenia was associated with poor clinical outcome in COVID-19 i.e. increased LOS, ICU admission and/or death. The median LOS was 5 days in the lymphopenic group and 4 days in the non lymphopenic group and this did not reach significance (p < 0.061;Fig. 3). We found that lymphopenia was associated with a significant increase in ICU admission (p < 0.022) and death (p < 0.0016).

### Effect of steroids administered upon admission on clinical outcomes

We then examined the effect of the administration of corticosteroids, both pre-admission and at the time of admission, on clinical outcomes. This was pertinent given that during the study period dexamethasone administration was standard of care for patients admitted to hospital for the management of COVID-19 respiratory failure. A small proportion of patients received treatment for COVID-19 prior to admission at a hospital study site. These included JAK inhibitors e.g. Baracitinab (N = 4), antivirals e.g. Molnupiravir (N = 1), Remdesivir (N = 18) and IL-6 receptor inhibitors e.g.Tocilizumab (N = 1), Sarilumab (N = 1) and/or synthetic monoclonal antibodies e.g. Sotrovimab (N = 27).

**Figure 4:**
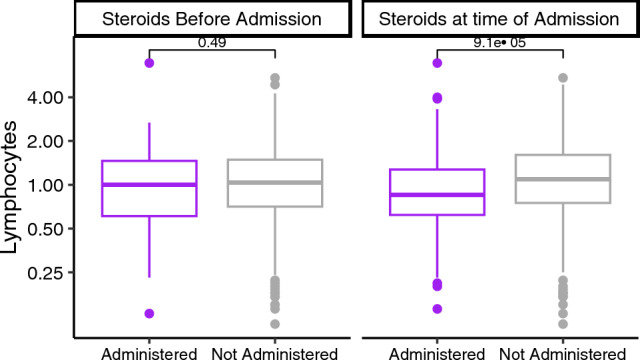
Lymphocyte counts and use of corticosteroids on admission. Patients administered steroids on admission had a statistically lower lymphocyte count compared to patients on steroids before admission. Grey = steroid not administered and purple = steroids administered before or at admission.

There was no difference in the lymphocyte counts of patients who received corticosteroids prior to admission, compared to those that had not (p = 0.49;Fig. 4). Patients who received corticosteroids on admission had lower lymphocyte counts than those who did not (p < 0.001). Additionally, patients who received corticosteroids at the time of admission were more likely to be admitted to ICU (Relative Risk = 2.45, 95%CI = 1.54–3.89). We investigated if the administration of corticosteroids in COVID-19 patients who were lymphopenic resulted in poorer outcomes. In patients given corticosteroids at the time of admission, the presence of lymphopenia did not result in significantly higher rates of ICU admission compared to those who were non-lymphopenic (RR = 1.08, 95%CI = 0.6–1.96) suggesting corticosteroid use in lymphopenic patients was not associated with worse clinical outcomes.

### Lymphopenia and laboratory parameters

**Figure 5:**
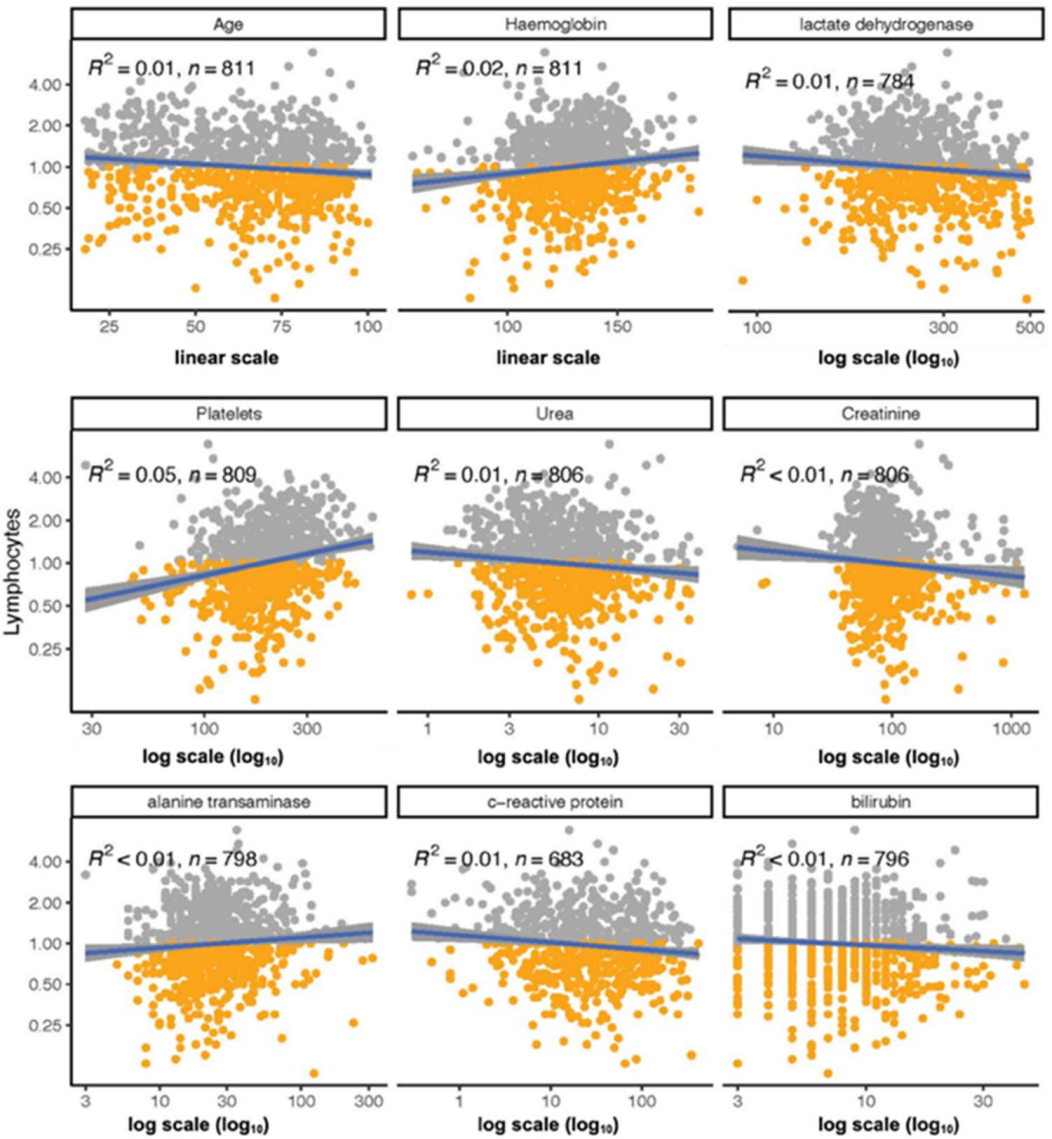
Lymphocyte counts and demographic and laboratory variables. There was significant correlation between lymphopenia and age, hemoglobin (hb), platelet count (plt), urea, c-reactive protein (crp) creatinine, bilirubin (bili), alanine transaminase(alt), and lactate dehydrogenase (ldh). Grey = non-lymphopenic; yellow = lymphopenic.

**Figure 6:**
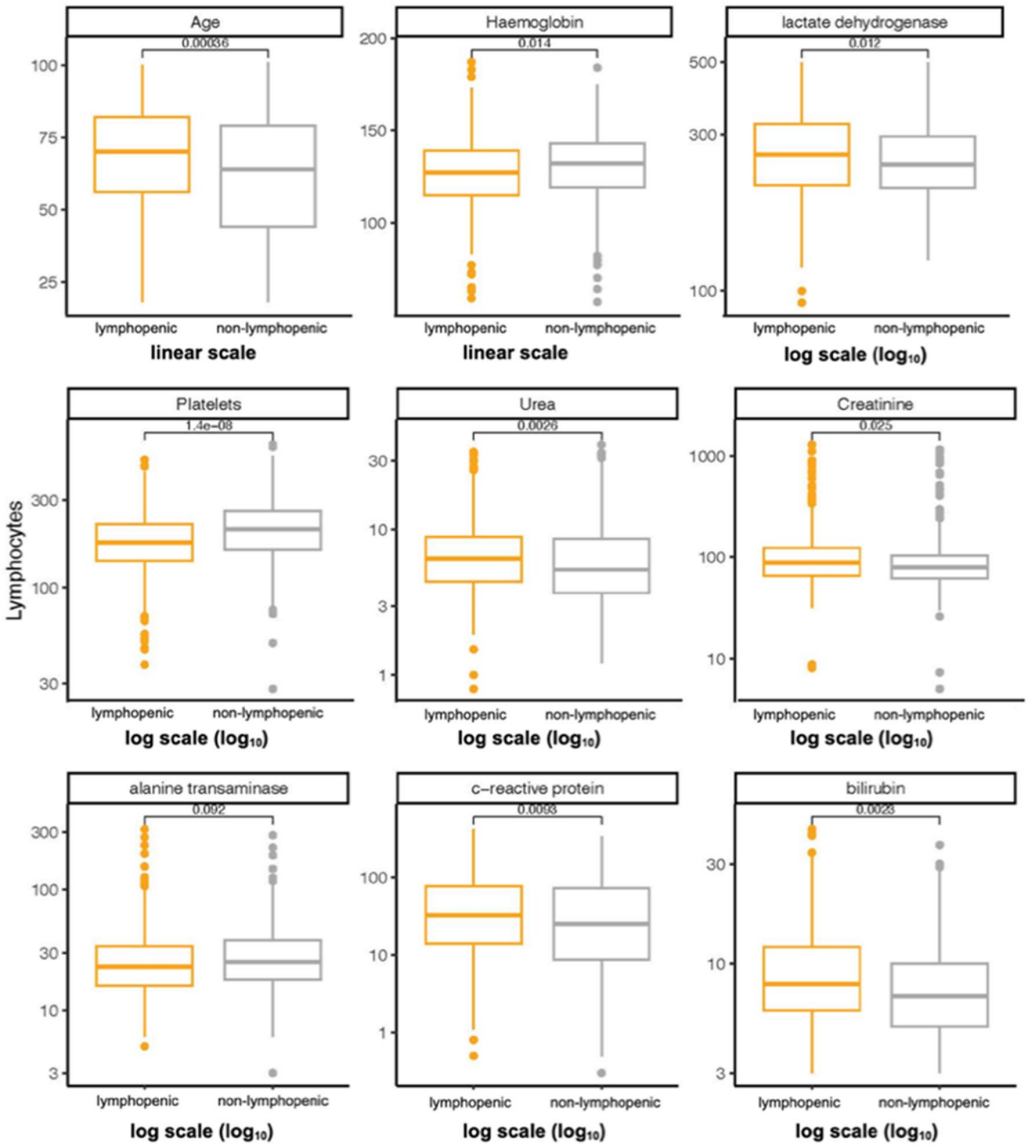
Differences in demographic and laboratory variables between lymphopenic and non-lymphopenic groups. Significant differences in age, c-reactive protein (CRP), haemoglobin (hb), lactate dehydrogenase (ldh), platelet count (plt), urea, creatinine (creat), bilibrubin (bili) and alanine transaminase (alt) are demonstrated. Grey = non-lymphopenic; yellow = lymphopenic.

Absolute lymphocyte counts were positively correlated with haemoglobin (R^2^ = 0.02), platelet count (R^2^ = 0.05) and the liver enzyme ALT (R^2^ < 0.01; see Fig. 5). Lymphocyte counts were negatively correlated with CRP (R^2^ = 0.01) and LDH (R^2^ = 0.01), as well as creatinine (R^2^ < 0.01) and urea (R^2^ = 0.01) and bilirubin (R^2^ < 0.01; Fig. 5). There was a negative correlation between lymphocyte counts and age (Fig. 5). There was no correlation between lymphocyte counts and neutrophils, AST, lipase, ferritin, lactate, and D-dimer (data not shown). After accounting for multiple comparisons, correlations between the absolute lymphocyte count and platelet count, haemoglobin, age, CRP, urea and LDH remained statistically significant, while those for creatine, bilirubin and the liver enzyme ALT did not. Additionally, for each of the six laboratory parameters that significantly correlate to the lymphocyte counts we confirmed that the median values for these analytes were also significantly different in lymphopenic hosts compared to non-lymphopenic hosts (Fig. 6).

### Multivariate analysis for clinical outcome

We conducted multivariate regression modelling to determine if lymphopenia was an independent predictor of poor clinical outcomes (Table 2). This analysis determined that lymphopenia was not an independent predictor of hospital LOS, ICU admission or death. The hospital LOS was independently predicted by age, haemoglobin, measures of CRP and LDH, use of post-admission corticosteroids and presence of chronic liver disease (Table 2). Independent predictors of ICU admission were age, CRP, LDH and diabetes as well as corticosteroid use prior to admission. Age, presence of diabetes and CRP were independent predictors of death.

**Table 2:** Independent predictors of hospital length of stay, ICU admission and death.

Measures of outcome	Clinical variables	Model coefficient^a^	p-value
Length of stay	Age	0.07	< 0.01
	Haemoglobin	− 0.05	< 0.01
	Steroids during stay	1.79	< 0.01
	C-reactive protein (log_10_)	1.31	0.01
	Presence of chronic liver disease	− 3.86	0.02
	Lactate dehydrogenase (log_10_)	5.31	0.02
ICU admission	C-reactive protein (log_10_)	1.41	< 0.01
	Steroids before admission (yes)	1.49	< 0.01
	Lactate dehydrogenase (log_10_)	3.60	< 0.01
	Age	− 0.02	< 0.01
	Diabetic (yes)	0.70	0.02
Death	C-reactive protein (log_10_)	2.04	< 0.01
	Age	0.06	< 0.01
	Diabetic (yes)	1.05	0.01

Clinical variables identified in our multivariate model as independent predictors of length of stay in hospital, ICU admission and death. All included variables have significant p-values (p < 0.05).

^a^Note that the model coefficient gives an estimate for how one unit of change in the clinical variable alters either (a) the prediction of the outcome measure (for Length of Stay) or (b) the odds of the outcome being true (ICU admission or death).

## Discussion

In this state-wide, prospective observational COVID-19 study, we found that lymphopenia was over-represented in males, those aged > 65 years and patients with hypertension which are all known predictors of poor outcomes in COVID-19. Furthermore, the death rate was higher in lymphopenic patients as compared to non-lymphopenic. There have been several previous reports that associate low lymphocyte counts with adverse clinical outcomes including mortality in COVID-19^3,4,6,17–20^. Severe COVID-19 and associated death have been correlated with increased age, male sex, black and minority ethnic groups and medical co-morbidities^21,22^. These medical conditions include but are not limited to haematological malignancy, bone marrow and solid organ transplant, primary and secondary immunodeficiency, asthma and other chronic lung diseases, chronic diseases of the kidney and liver^21,22^.

We found that COVID-19 lymphopenia was associated with hypertension consistent with previous studies^23,24^. Interestingly, we report a negative correlation between lymphopenia and the presence of asthma, possibly suggesting asthma may be a protective factor against low lymphocyte counts in COVID-19^25^ but this requires further validation. The proportion of patients who received two or three doses of COVID-19 vaccine did not differ significantly across the lymphopenic and non-lymphopenic groups, suggesting vaccination was a not a co-founder for lymphopenia.

We provide additional independent verification that the median lymphocyte count is lower in patients requiring ICU admission (0.88 versus 1.04 × 10^6^/L; p = 0.022; Fig. 3) and in those who died (0.74 versus 1.05 × 10^6^/L; p = 0.0016; Fig. 3). We investigated if post-admission steroids use was associated with worse outcomes in lymphopenic patients. Patients who were lymphopenic on admission and subsequently received corticosteroids were not more likely to require ICU admission compared to non-lymphopenic counterparts (RR = 1.08), an important finding given the high use of corticosteroid use in the management of COVID-19^6,24^.

We found that the absolute lymphocyte count, positively correlated to both haemoglobin level and platelet count, suggesting that low measures across these cell lineages may be the result of decreased bone marrow output, increased peripheral consumption/destruction, or a combination of both. For the first time we report that absolute lymphocyte count is inversely associate with urea, creatinine, bilirubin, ALT linked to liver and kidney impairment, but only urea remained as a statistically significant analyte after multiple comparisons.

While prior studies identified an association between lymphopenia and poor clinical outcome, this work builds on those findings by investigating whether lymphopenia is an independent predictor for adverse outcomes in COVID-19, an aspect not previously explored. Our findings reveal that lymphopenia was a correlate but not an independent predictor of hospital LOS, ICU admission and death. Age and diabetes emerged as independent predictors of all three adverse clinical outcomes. Moreover, CRP independently predicted all three outcomes. LDH was an independent predictor of longer LOS and ICU admission but not death. Chronic liver disease and haemoglobin only independently predicted longer LOS.

Our work has several limitations. Our data derive from one region of Australia, and it is important to validate and compare these findings in different geographical locations. Furthermore, due to the unavailability of data on ethnicity, we could not explore its role in our findings. Additionally, this study did not capture dynamic changes in lymphocytes or other variables over time, including during recovery. Further dissecting the dynamics of lymphocyte recovery over time and in relation to Post Acute Sequalae of COVID-19 would be of interest. As this study utilised standard of care clinical data, extended viral sequencing to determine the SARS-CoV-2 viral subtype, including variants of concern was not performed. However, extensive molecular virology performed in Australian populations during the same time-period provides evidence that the 649 patients from our cohort who tested positive from January 2022 are highly likely to have been infected by the BA.1 Omicron variant^26^. This study focuses on lymphopenia in COVID-19 sepsis, however lymphopenia is a common feature of other forms of bacterial and viral sepsis^2^. In non-COVID-19-related sepsis, lymphopenia has been correlated to adverse clinical outcomes including an elevated risk of 28-day mortality^27^. A study patients with sepsis admitted to the ICU found that survivors exhibited a higher proportion of lymphocytes, particularly T cells and NK cells, compared to non-survivors^28^. Additionally, patients with severe community acquired pneumonia displayed low lymphocyte counts, with a significant association of low CD4^+^ T cells and high risk of 30-day mortality^29^. Therefore, further investigations are warranted to determine whether the association of lymphopenia with demographics or co-morbidities observed in this study are replicable in other forms of sepsis, unrelated to COVID-19.

Sepsis is a global health priority, and remains the leading cause of death in ICUs, even in resource-rich settings such as Australia. There is an imperative to further understand distinct subgroups of septic patients, such as those with lymphopenia, who are at the highest risk of serious morbidity and mortality. Our work and others have demonstrated that lymphopenia clearly associates with poor outcomes such as admission to ICU and mortality, despite other variables being more powerful, independent predictors. This highlights a significant need to delineate the cellular and molecular drivers of lymphopenia in COVID-19 and other forms of sepsis. Indeed host-directed therapies such as IL-7 for the reversal of lymphopenia^30^ and immune checkpoint inhibitors for the reversal of T cell anergy^31^ are currently under investigation for use in lymphopenic sepsis. Overall, identifying novel therapeutic targets to curb excess sepsis mortality in those at highest risk is a research priority.

In conclusion, in critically ill adults with severe COVID-19 requiring ICU care, lymphopenia is associated with older age, male, and presence of hypertension and laboratory biomarkers of inflammation, kidney, and liver damage. We independently verify that in univariate analysis lymphopenia correlates to ICU admission and death, however, demonstrate for the first time that lymphopenia is not a statistically independent predictor for these adverse clinical outcomes.

### Supplementary Information

Supplementary Information.

## Data Availability

The dataset used and analysed during the current study are available from the corresponding author on reasonable request.

## Abbreviations

ENTER-COVID: Epidemiology of hospitalized in-patient admissions following planned introduction of epidemic SARS-CoV-2 to highly vaccinated COVID-19 naïve population
ICU: Intensive care unit
N: Number of patients
AST: Aspartate aminotransferase
ALT: Alanine transaminase
LDH: Lactate dehydrogenase
CRP: C-reactive protein
LOS: Length of stay
IQR: Interquartile ranges
RR: Relative risk
IL-7: Interleukin-7
ISARIC: International Severe Acute Respiratory and Emerging Infection Consortium
RAH: Royal Adelaide Hospital
NHMRC: National Health and Medical Research Council
HREC: Human Research Ethics Committee
