# Supplementary material for: Defining the correlates of lymphopenia and independent predictors of poor clinical outcome in adults hospitalized with COVID-19 in Australia
Source: Sci Rep. 2024 May 15;14:11102. doi: 10.1038/s41598-024-61729-5 (PMC11096393; doi:10.1038/s41598-024-61729-5)
Supplement: Supplementary file 1 — Supplementary Information. [file 41598_2024_61729_MOESM1_ESM.docx]

**Supplementary Materials:**

**Supplementary Table 1:** Upper limit threshold for laboratory analytes in the ENTER-COVID dataset to conduct downstream statistical analysis.

| **Laboratory Analyte** | **Upper limit** |
| --- | --- |
| haemoglobin | 360 g/dl |
| platelet count | 1000 x 10^9/L |
| neutrophils | 16 x 10^9/L |
| urea | 100 mmol/L |
| creatinine | 1500 mmol/L |
| bilirubin | 50 mmol/L |
| alanine transaminase | None |
| aspartate transaminase | 1000 U/L |
| lactate dehydrogenase | 500 U/L |
| lactate | 2 mmol/L |
| d-dimer | 10 mg/L |
| C-reactive protein | 1000 mg/L |
| ferritin | 100000 ng/mL |
| lipase | None |

**Supplementary Figure 1:** Median lymphocyte counts and tuberculosis, malignant neoplasm and HIV. Patients with malignant neoplasm had a statistically lower lymphocyte count. Grey= non-lymphopenic and red= lymphopenic patients
